# Transcranial Magnetic Stimulation and its Efficacy in Alleviating Depressive Symptoms in Patients with Suicidal Ideation

**DOI:** 10.1192/j.eurpsy.2024.395

**Published:** 2024-08-27

**Authors:** A. Moleon, M. Martín-Bejarano, P. Alvarez de Toledo, I. Perez, J. Narbona, M. García-Ferriol, R. Perea, J. M. Oropesa, J. Torres

**Affiliations:** ^1^Instituto Andaluz de Salud Cerebral (IASC), Sevilla; ^2^Instituto Andaluz de Salud Cerebral (IASC), Huelva, Spain

## Abstract

**Introduction:**

Suicide is a global public health issue. According to the latest available data from the National Institute of Statistics, 4,003 people died by suicide in 2021, reaching a new historical high. Approximately 90% of suicide victims suffer from one or more severe psychiatric disorders, and there is a documented 20-fold higher risk of suicide in individuals with affective disorders compared to healthy subjects (Abdelnaim et al., 2020). Repetitive transcranial magnetic stimulation (rTMS) has been established as an effective alternative or complementary treatment option for patients with depressive disorders, but little is known about its effects on suicide risk.

**Objectives:**

To assess the efficacy of rTMS in reducing depressive symptoms in patients with suicidal ideation and behaviors.

**Methods:**

Population and Methods: A retrospective analysis was conducted on a sample of 28 psychiatric patients (23 females; mean age 49.36 ± 16.23) with suicidal ideation identified by item 3 (suicidality) of the Hamilton Depression Rating Scale (HDRS), who were treated with rTMS. All patients received a minimum of 30 sessions, consisting of the application of a high-frequency (>10Hz) or intermittent theta burst stimulation (TBS) over the left dorsolateral prefrontal cortex (DLPFC) at an intensity of 120% of the resting motor threshold (RMT), and repeated low-frequency pulses (1Hz) or continuous TBS over the right DLPFC with an intensity of 110% of the RMT.

**Results:**

Results: The results show a statistically significant improvement in depressive symptoms following rTMS intervention (p < 0.001). Furthermore, remission was observed in 46% of the sample (HDRS < 8).

**Conclusions:**

Discussion: In line with recent studies (Abdelnaim et al., 2020; Hines et al., 2022) and systematic reviews (Cui et al., 2022; Bozzay et al., 2020) on suicidal ideation in the context of psychiatric disorders, the findings of this study demonstrated that rTMS achieved satisfactory results in reducing depressive symptoms and suicidal ideation.

Conclusions: This clinical study indicates preliminary promise for the prevention of suicidal acts and underscores the need for more detailed and specific research on rTMS in the field of suicide.

Keywords: rTMS, neuromodulation, depression, suicide.

**Disclosure of Interest:**

None Declared

